# Herpes simplex virus infections among rural residents in eastern China

**DOI:** 10.1186/1471-2334-11-69

**Published:** 2011-03-18

**Authors:** Haijiang Lin, Na He, Meifang Su, Jifu Feng, Li Chen, Meiyang Gao

**Affiliations:** 1 Department of Epidemiology, School of Public Health, Fudan University; Key Laboratory of Public Health Safety (Fudan University), Ministry of Education, PR China; 2 Taizhou City Center for Disease Control and Prevention, Zhejiang Province, PR China; 3 Yuhuan County Center for Disease Control and Prevention, Zhejiang Province, PR China

**Keywords:** Seroprevalence, Herpes simplex virus, Hepatitis B virus, Community residents, Eastern China

## Abstract

**Background:**

Herpes simplex virus (HSV) has two types: HSV-1 and HSV-2. Both infect epithelial cells and establish latent infections in neurons causing an infection that persists for life. Information on age- and gender-specific seroprevalence of HSV-1 and HSV-2 is valuable for understanding HSV transmission dynamics and designing population-based prevention and intervention programs for HSV. However, such information is not available for China.

**Methods:**

Cryopreserved serum samples of all subjects aged 5 to 60 years from two randomly selected rural villages in Zhejiang province in Eastern China who had participated in the China national seroepidemiological survey of hepatitis B virus (HBV) infection conducted in 2006 were tested. Seroprevalence of HSV-1 and HSV-2 infections were determined by type-specific IgG antibody tests using an ELISA technique. Their 95% confidence intervals adjusted for the sampling fraction were calculated according to the Clopper-Pearson method.

**Results:**

A total of 2,141 residents participated in the survey, with a response rate of 82.3%. HSV-1 seroprevalence was 92.0% overall, 89.1% for males and 94.2% for females. HSV-1 seroprevalence was 61.6% among children aged 5-9 years, 90.3% among 25-29 years, and nearly 100% among those aged > = 40 years. HSV-2 seroprevalence was 13.2% overall, 10.5% for males and 15.3% for females. No children aged 5-14 years were HSV-2 positive, and HSV-2 seroprevalence was 7.1% among 15-19 years and peaked at 24.3% among those aged 45-49 years. Neither HSV-1 nor HSV-2 infections were significantly different by gender. About 11.8% of study subjects were co-infected with both types of HSV. Among 549 participating couples, 8.6% were HSV-1 serodiscordant and 11.8% were HSV-2 serodiscordant. No one tested positive for HIV. The overall prevalence of HBsAg was 16.2%, 16.9% for males and 15.4% for females.

**Conclusions:**

HSV-1 was highly prevalent among all rural residents aged between 5-60 years in Eastern China, whereas HSV-2 was prevalent among sexually active people. HSV-1 and HSV-2 have different transmission modes and dynamics. Future HSV prevention and control programs in China should be type specific.

## Background

Herpes simplex virus (HSV) belongs to subfamily alpha of herpesvirinae and has two types: HSV-1 and HSV-2. Both infect epithelial cells and establish latent infections in neurons, causing an infection that persists for life. Although HSV-1 and HSV-2 infections are generally asymptomatic, both viruses can cause a wide spectrum of clinical manifestations. HSV-1 is classically associated with oropharyngeal lesions transmitted horizontally in childhood, causes recurrent attacks of "fever blisters", and has been shown to account for an increasing proportion of primary genital herpes, especially in women [1]. It is also the leading cause of sporadic encephalitis in the United States and several other countries [2]. HSV-2 primarily infects the genital mucosa and is the main cause of genital herpes. HSV-2 infection is acquired primarily through contact with infected genital secretions and this strong association, provides a useful marker for sexual behavior [3]. Further, it is widely implicated as an important cofactor for HIV acquisition [4,5]. HSV-2 is one of the most common causes of neonatal herpes and also can cause encephalitis in the immunocompromised host [6]. Meanwhile, clinical manifestations of a chronic HSV infection (HSV-1 or HSV-2) among HIV/AIDS patients have been regarded by the World Health Organization (WHO) as an important presentation defining the disease progression of HIV/AIDS [7].

Both HSV-1 and HSV-2 infections are known to be common throughout the world [8]. However, China currently has no estimates of the prevalence of HSV-1 infection and only limited estimates of HSV-2 infection among selected subpopulations [9-16]. Additionally there have been no studies that have ascertained age- and gender-specific seroprevalence of HSV-1 and HSV-2 in the general population in China. Such information is valuable for understanding HSV transmission dynamics and designing type-specific, population-based prevention and intervention programs for HSV. To provide these estimates, we conducted a seroepidemiologic survey of HSV among rural community residents in Zhejiang province, Eastern China. Given the association of HSV-2 infection with sexual behavior and HIV acquisition, we also tested for HIV infection in this study. Furthermore, since Hepatitis B virus (HBV) infection is endemic in China and is also associated with sexual contact and classified by WHO as a sexually transmitted disease, we also reported the seroprevalence of HBV infection among the study participants.

## Methods

### Study Sample

In 2006, the Chinese Center for Disease Control and Prevention (Chinese CDC) organized and conducted a national seroepidemiological survey of hepatitis B virus (HBV) infection (China Ministry of Health, April 21, 2008). As part of this study, two out of seventy-two villages in the rural community of Zhejiang province in Eastern China were randomly selected using a cluster sampling strategy and all local permanent residents between the ages of 5 to 60 years were recruited to participate. Those 18 years or older provided consent for themselves and parental consent was obtained for those less than 18. The consent form clearly illustrated the background, importance and significance of the study, and documented that future use of stored blood samples would be approved by the relevant Institutional Review Board (IRB). The initial study was approved by the IRB of the Chinese CDC. From these two villages, a total of 2,600 eligible residents were approached and 2,141 (82.3%) participated in the survey. Table 1 presents age- and gender-specific participation rates. The overall participation rate was not significantly different by gender (Chi-squared test, **χ**^2 ^= 0.01; *P *= 0.911) but significantly different by age groups (Chi-squared test, **χ**^2 ^= 45.78; *P *< 0.001). Venous blood was collected from all participants by professional nurses using disposable sterile needles and tubes. The blood was centrifuged, and the serum was frozen in 500-μL aliquots.

**Table 1:** Age- and gender-specific seroprevalence of HSV-1 and HSV-2 among rural residents in Eastern China

	Male (n = 1065)				Female (n = 1076)				Total (n = 2141)			
			
			HSV-1	HSV-2			HSV-1	HSV-2			HSV-1	HSV-2
									
Age (years)	Tested/Eligible	%*	%** (95% CI)	%** (95% CI)	Tested/Eligible	%*	%** (95% CI)	%** (95% CI)	Tested/Eligible	%*	%** (95% CI)	%** (95% CI)
5-	75/106	70.8	59.8 (20.9-98.8)	0	40/54	74.1	64.9 (46.4-83.5)	0	115/160	71.9	61.6 (27.3-95.8)	0
10-	64/84	76.2	78.0 (47.6-100.0)	0	45/74	60.8	78.3 (37.8-100.0)	0	109/158	69.0	78.1 (75.2-81.1)	0
15-	89/109	81.7	76.5 (68.9-84.0)	5.4 (0-33.5)	78/97	80.4	87.5 (20.0-100.0)	8.9 (0-18.6)	167/206	81.1	81.6 (48.4-100.0)	7.1 (0-19.2)
20-	93/120	77.5	81.9 (43.2-100.0)	8.9 (0-64.1)	101/121	83.5	86.1 (64.5-100.0)	12.0 (0-48.7)	194/241	80.5	84.0 (78.0-90.0)	10.5 (0-54.5)
25-	112/134	83.6	86.5 (64.5-100.0)	5.3 (0-14.6)	135/155	87.1	93.3 (58.3-100.0)	13.3 (0-37.0)	247/289	85.5	90.3 (58.3-100.0)	9.7 (0-30.2)
30-	137/157	87.3	90.4 (72.2-100.0)	10.2 (2.4-18.0)	152/182	83.5	96.7 (94.7-100.0)	13.1 (5.0-21.3)	289/339	85.3	93.8 (82.3-100.0)	11.8 (11.5-12.0)
35-	144/163	88.3	93.7 (85.2-100.0)	13.8 (0-34.6)	154/182	84.6	95.5 (81.4-100.0)	16.2 (0-77.7)	298/345	86.4	94.6 (82.8-100.0)	15.1 (0-57.7)
40-	118129	91.5	97.4 (89.6-100.0)	13.6 (6.8-20.4)	129/153	84.3	98.4 (97.7-99.2)	17.8 (0-38.2)	247/282	87.6	98.0 (94.5-100.0)	15.9 (2.7-29.0)
45-	89/113	78.8	96.7 (60.5-100.0)	23.7 (0-100.0)	97/114	85.1	98.0 (76.9-100.0)	24.9 (0-60.5)	186/227	81.9	97.4 (68.7-100.0)	24.3 (0-84.8)
50-	82/106	77.4	98.7 (87.7-100.0)	8.5 (0-52.1)	101/120	84.2	98.1 (69.7-100.0)	16.1 (0-80.7)	183/226	81.0	98.4 (88.4-100.0)	12.5 (0.3-24.8)
55-	62/71	87.3	98.4 (76.2-100.0)	16.1 (4.7-37.0)	44/56	78.6	97.8 (71.8-100.0)	16.1 (0-63.0)	106/127	83.5	98.1 (97.1-99.1)	16.1 (7.1-25.2)
Total	1065/1292	82.4	89.1 (70.0-100.0)	10.5 (10.4-10.5)	1076/1308	82.3	94.2 (94.0-94.5)	15.3 (0-33.3)	2141/2600	82.3	92.0 (84.2-99.8)	13.2 (3.6-22.7)

*: participation rate; **: seroprevalence

For the present secondary data analysis, the cryopreserved serum samples from these participants were tested to ascertain age- and gender-specific seroprevalence of HSV-1 and HSV-2 infections. The testing was performed anonymously and the serological results of HSV-1 and HSV-2 infections were not linked to the participants. The protocol for the present serological study was reviewed and approved by the Institutional Review Board (IRB) of Fudan University, Shanghai, China.

### Blood testing

HSV Antibody Testing: HSV type-specific IgG antibody was tested using an enzyme-linked immunosorbent assay (ELISA) technique (HerpeSelect 1 ELISA IgG Kit and HerpeSelect 2 ELISA IgG Kit, Focus Technologies, CA, USA). All tests were performed by two experienced technicians from the key laboratory of the leading institution, according to the manufacturers' standard protocols. Each plate run included duplicate Cut-off Calibrators and four controls including a High Positive Control, a Low Positive Control, a Negative Control and a Blank Control. Results were reported as index values relative to the Cut-off Calibrator. To calculate index values, specimen optical density (OD) values were divided by the mean of the duplicate Cutoff Calibrator absorbance values. An index value of >1.10 was reported as seropositive for HSV-1 or HSV-2, and those with index values < 0.9 were recorded as negative, and those with index values between 0.9 and 1.1 were recorded as equivocal and were retested. Those retested and found to have index values >1.1 were recorded as positives. Those samples in which results remained equivocal were retested using another ELISA technique (HerpeSelect 1 ELISA IgG Kit and HerpeSelect 2 ELISA IgG Kit, Euroimmun, Lübeck, Germany). Only 34 (1.6%) samples, 32 for HSV-1 and 2 for HSV-2 in this study were recorded as equivocal on the first test but not equivocal on re-testing. The results of the 34 samples retested were all negative.

#### HIV and HBV testing

Anti-HIV IgG antibody and HBV surface antigen (HBsAg) were tested using an ELISA technique (Wantai Biological Pharmacy Enterprise Co., Beijing, China). All tests were performed according to the manufacturer's standard protocols.

### Data Analysis

Seroprevalence of HSV-1 and HSV-2 as well as their 95% confidence intervals (CIs) adjusted for the sampling fraction were calculated and tabulated by stratifying on age and gender, according to the Clopper-Pearson method using the software package Stata 11.0 (StataCorp, Texas, USA). Seroprevalence of HBsAg was also calculated by age and gender.

## Results

### HSV-1 Seroprevalence

**Figure 1:**
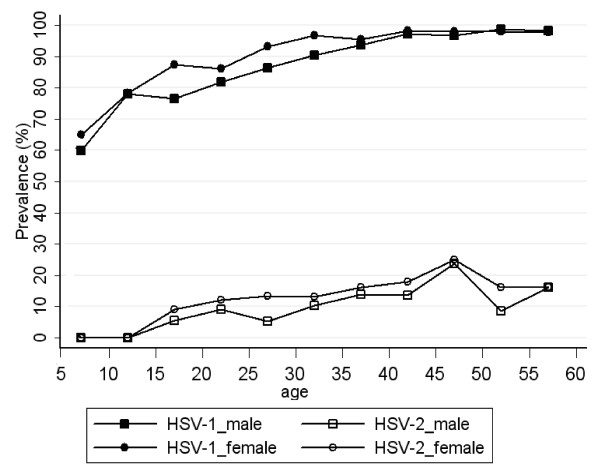
**Age- and gender-specific seroprevalence of HSV-1 and HSV-2 among rural residents in Eastern China**.

As shown on Table 1, the overall HSV-1 seroprevalence was 92.0% in the study sample, and was comparable by sex, 89.1% for males and 94.2% for females (Figure 1). High levels of HSV-1 infection were found very early in childhood, with a seropositivity of 61.6% among children aged 5-9 years. HSV-1 seroprevalence increased to 90.3% among those aged 25 to 29 years and gradually increased up to nearly 100% among those aged over 40 years.

### HSV-2 Seroprevalence

The overall HSV-2 seroprevalence was 13.2%, and was also comparable between males and females at each age group, 10.5% for males and 15.3% for females (Table 1 and Figure 1). No participants 5 to 14 year old in this sample were infected with HSV-2. HSV-2 seroprevalence was first detected among the 15 to 19 year old age group (7.1%) and then gradually rose to a rate of 24.3% among those aged at 45 to 49.

### HSV-1 and HSV-2 Co-infections

Approximately 11.8% (252/2,141) of participants were co-infected with both types of HSV. Co-infections were more common among females (13.6%) than males (10.0%). There were a total of 549 participating couples. The mean age was 40.14 years (standard deviation: 9.77) and the median age was 39.37 for husbands. The mean age was 37.82 years (standard deviation: 9.30) and the median age was 36.95 for wives. Among these couples, 498 couples (90.7%) were seroconcordant HSV-1 positives, 47 couples (8.6%) were HSV-1 serodiscordant and only 4 couples (0.7%) were seroconcordant HSV-1 negatives; whereas 44 couples (8.0%) were seroconcordant HSV-2 positives, 65 couples (11.8%) were HSV-2 serodiscordant and 440 couples (80.1%) were seroconcordant HSV-2 negatives.

### HIV and HBsAg seroprevalence

No one tested positive for HIV. The overall prevalence of HBsAg was 16.2%, 16.9% for males and 15.4% for females. The age-specific prevalence of HBsAg among males was 5.3%, 6.4%, 14.6%, 14.0%, 19.8%, 16.8%, 17.6%, 27.1%, 16.3%, 17.3%, 23.3%, respectively, with age groups corresponding to the eleven five-year categories shown in Table 1 (data not shown). Similarly, the age-specific prevalence of HBsAg among females was 5.0%, 6.7%, 14.3%, 13.9%, 12.5%, 15.1%, 20.0%, 20.9%, 15.5%, 11.8%, 21.7%, respectively, for the same eleven age groups (data not shown).

## Discussion

To our knowledge, this is the first study to document age- and gender-specific seroprevalence of both types of HSV in a general population sample in China. We observed a very high level of HSV-1 infection (92.0%) and a relatively low level of HSV-2 infection (13.2%). These observations are consistent with those in other countries [8], but are significant, as HSV-1 prevalence has never been reported in China. Limited studies conducted in China have demonstrated HSV-2 prevalence as high as 70% among female sex workers and as low as 5% among general male rural migrants [10,13-15], with rates typically higher in women than men. Our gender-specific HSV-2 prevalence was 10.5% for males and 15.3% for females and was found to be higher than that among market vendors (6.4% for males and 12.0% for females) in Fuzhou, the capital city of Fujian province and among male migrants in Shanghai (5.5%) [9,10,15]. Such differences might be due to differences in the characteristics of the study subjects. For instance, the participants in the present study were local permanent residents while those in the referred two studies were rural-to-urban migrants, with possible greater opportunity for infection.

Differences in the age distributions between HSV-1 and HSV-2 infections in the present study provide evidence to the supposition that HSV-1 and HSV-2 have different transmission modes and dynamics [17,18]. While HSV-1 is highly epidemic and ubiquitous for all age groups, HSV-2 infection is most likely to occur among sexually active people. Our findings suggest that future HSV prevention and intervention programs should be type-specific. Meanwhile, we found that a substantially higher proportion of couple participants were seroconcordant HSV-1 positive (90.7%) versus HSV-1 serodiscordant (8.6%) than seroconcordant HSV-2 positive (8.0%) versus HSV-2 serodiscordant (11.6%). These findings have two possible explanations. First, HSV-1 prevalence was found to be very high among the general adult population. Therefore, the low proportion of HSV-1 serodiscordance among couples might be due to the low prevalence of HSV-1 negative; Second, HSV-1 can be transmitted by both sexual and nonsexual contacts whereas HSV-2 is almost always transmitted by sexual contacts. Therefore, HSV-1 transmission between couples is more likely to occur than HSV-2. Given that HSV-2 is predominantly sexually transmitted and most couples in China do not use condoms for marital sex [19], marital HSV-2 transmission could be a concern for future STI prevention and control programs in Eastern China.

The limitations of the serological tests for HSV-1 and HSV-2 should be noted. In this study, Focus Diagnostics ELISAs were used for HSV-1 and HSV-2 serum antibody detection in order to determine cumulative lifetime exposure to infection. Previous validation studies have shown high sensitivity but relatively poorer specificity for HSV-1 and HSV-2 Focus Diagnostics ELISAs using index values of 1.10 when compared to the golden standard HSV western blot [20,21]. This relatively poor specificity could yield false positives, which might have leaded to an overestimation of the prevalence of HSV-1 and HSV-2 [20,21]. However, this study was based on a large sample size, thus the age- and gender-specific prevalence patterns for HSV-1 and for HSV-2 should be negligibly affected.

In addition, no one in the present study was positive for HIV, suggesting a low HIV epidemic at the population level. On the other hand, HBV infection was still prevalent in this population especially among those aged over 15 years. As HBV infection could be sexually transmitted and because both HIV and HBV have severe health outcomes, they are not ignorable for any successful STI prevention and intervention programs in these communities.

## Conclusions

HSV-1 infection is highly prevalent among all age groups whereas HSV-2 infection is most likely to occur among sexually active people in Eastern China. HSV-1 and HSV-2 have different transmission modes and dynamics in the population, and need type specific prevention and control programs.

## Competing interests

The authors declare that they have no competing interests.

## Authors' contributions

HL contributed to conceptual design, data collection, cleaning and analysis, and preparation of the manuscript. NH contributed to conceptual design, interpretation, and preparation of the final manuscript. MS and JF participated in the implementation of the study and contributed to subject recruitment, sample and data collection. Laboratory sample processing was performed by LC and MG. All authors had access to all data and contributed to the final draft of the paper. All authors read and approved the final manuscript.

## Pre-publication history

The pre-publication history for this paper can be accessed here:

http://www.biomedcentral.com/1471-2334/11/69/prepub
